# Genetic variability, community structure, and horizontal transfer of endosymbionts among three Asia II‐*Bemisia tabaci* mitotypes in Pakistan

**DOI:** 10.1002/ece3.6107

**Published:** 2020-02-12

**Authors:** Jorge R. Paredes‐Montero, Muhammad Zia‐Ur‐Rehman, Usman Hameed, Muhammad Saleem Haider, Hans‐Werner Herrmann, Judith K. Brown

**Affiliations:** ^1^ School of Plant Sciences University of Arizona Tucson AZ USA; ^2^ Facultad de Ciencias de la Vida Escuela Superior Politécnica del Litoral (ESPOL) Guayaquil Ecuador; ^3^ Institute of Agricultural Sciences University of the Punjab Lahore Pakistan

**Keywords:** cotton leaf curl disease, cryptic species, horizontal transfer, microbiome, sweet potato whitefly

## Abstract

Endosymbionts associated with the whitefly *Bemisia tabaci* cryptic species are known to contribute to host fitness and environmental adaptation. The genetic diversity and population complexity were investigated for endosymbiont communities of *B. tabaci* occupying different micro‐environments in Pakistan. Mitotypes of *B. tabaci* were identified by comparative sequence analysis of the mitochondria cytochrome oxidase I (mt*COI*) gene sequence. Whitefly mitotypes belonged to the Asia II‐1, ‐5, and ‐7 mitotypes of the Asia II major clade. The whitefly–endosymbiont communities were characterized based on 16S ribosomal RNA operational taxonomic unit (OTU) assignments, resulting in 43 OTUs. Most of the OTUs occurred in the Asia II‐1 and II‐7 mitotypes (*r* ^2^ = .9, *p* < .005), while the Asia II‐5 microbiome was less complex. The microbiome OTU groups were mitotype‐specific, clustering with a basis in phylogeographical distribution and the corresponding ecological niche of their whitefly host, suggesting mitotype‐microbiome co‐adaptation. The primary endosymbiont *Portiera* was represented by a single, highly homologous OTU (0%–0.67% divergence). Two of six *Arsenophonus* OTUs were uniquely associated with Asia II‐5 and ‐7, and one occurred exclusively in Asia II‐1, two only in Asia II‐5, and one in both Asia II‐1 and ‐7. Four other secondary endosymbionts, *Cardinium*, *Hemipteriphilus*, *Rickettsia*, and *Wolbachia* OTUs, were found at ≤29% frequencies. The most prevalent *Arsenophonus* OTU was found in all three Asia II mitotypes (55% frequency), whereas the same strain of *Cardinium* and *Wolbachia* was found in both Asia II‐1 and ‐5, and a single *Hemipteriphilus* OTU occurred in Asia II‐1 and ‐7. This pattern is indicative of horizontal transfer, suggestive of a proximity between mitotypes sufficient for gene flow at overlapping mitotype ecological niches.

## Graphical abstract

This manuscript describes the diversity of endosymbionts associated with *Bemisia tabaci* in Pakistan. It was shown for the first time that *B. tabaci* mitotypes and their associated secondary endosymbiont are geographically codistributed and that the distributions are consistent with climatic gradient niches, suggestive of ecologically associated whitefly–endosymbiont co‐adaptation.

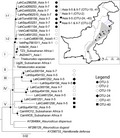

## 1 INTRODUCTION

The whitefly *Bemisia tabaci* (Gennadius) group (Brown, Frohlich, & Rosell, 1995; Gill & Brown, 2010) consists of five or more cryptic lineages (Brown, 2010; Brown et al., 1995; Lee, Park, & Lee, 2013; de Moya et al., 2019; Paredes‐Montero et al., 2019), some of which contain mitotype groups that are important plant pests and/or vectors of plant viruses (Jones, 2003). For more than fifty years, whitefly‐transmitted viruses in the genus *Begomovirus* (family Geminiviridae) have been recognized as important new or emergent pathogens in irrigated agroecosystems worldwide (Jones, 2003). The variable dynamics of begomovirus emergence and spread have been linked to phenotypic differences in whitefly mitotypes and their endosymbiont composition (Bedford, Briddon, & Brown, 1994; Brown & Czosnek, 2002; Gilbertson, Batuman, Webster, & Adkins, 2015; Idris, Smith, & Brown, 2001). Endosymbionts of whiteflies and other hemipterans affect their biology and fitness (Baumann, 2005; Brumin, Kontsedalov, & Ghanim, 2011; Caballero, 2007; Himler et al., 2011). Although the molecular basis for whitefly‐begomovirus transmission specificity is still poorly understood, several endosymbiont‐ and whitefly‐encoded proteins have been implicated in whitefly‐mediated transmission (Ghosh, Bouvaine, & Richardson, 2018; Gottlieb et al., 2010; Gotz et al., 2012; Himler et al., 2011; Kliot, Cilia, Czosnek, & Ghanim, 2014; Morin, Ghanim, Sobol, & Czosnek, 2000; Su et al., 2013).

The primary endosymbiont (P‐endosymbiont), *Candidatus* (*Ca.*) Portiera Aleyrodidarum (hereafter, *Portiera*), is vertically transmitted and has co‐evolved with its whitefly hosts (Baumann, 2005) by contributing essential amino acids and vitamins not present in the phloem sap (Baumann, 2005; Luan et al., 2015; Santos‐Garcia et al., 2012). Cospeciation occurs commonly among insects that feed in the phloem or xylem (Thao & Baumann, 2004), including aphids (Clark, Moran, Baumann, & Wernegreen, 2000), leafhoppers (Takiya, Tran, Dietrich, & Moran, 2006), mealybugs (Baumann & Baumann, 2005), and psyllids (Thao et al., 2000). However, secondary endosymbionts (S‐endosymbionts) are represented by a large number of diverse bacteria (Bing, Yang, & Zchori‐Fein, 2013; Gottlieb et al., 2006; Zchori‐Fein & Brown, 2002; Zchori‐Fein, Lahav, & Freilich, 2014), representing putative “major” and “minor” secondary endosymbionts. For *B. tabaci*, the secondary endosymbionts have been shown to confer thermal tolerance (Brumin et al., 2011; Russell & Moran, 2006) and resistance to parasitoid attack (Mahadav, Gerling, & Gottlieb, 2008). Thus far, two major secondary endosymbionts have been identified in *B. tabaci*, *Arsenophonus nasoniae,* or *Hamiltonella defensa* (hereafter, *Arsenophonus* and *Hamiltonella*, respectively). They are not considered to be as vital for survival of *B. tabaci*, as is the primary endosymbiont *Portiera*, but instead, serve as source of B vitamins (Santos‐Garcia et al., 2018), contribute to aspects of fitness, to combat abiotic and biotic environmental stress and at times to gain reproductive advantages, and many of them are known to be horizontally transmitted (Ghanim & Kontsedalov, 2009; Gonella et al., 2015; Kontsedalov et al., 2008; Raina et al., 2015; Santos‐Garcia et al., 2018; Zchori‐Fein et al., 2001). The minor secondary endosymbionts of *B. tabaci*, including *Ca.* Cardinium spp., *Ca.* Wolbachia spp. (Caballero, 2007), and *Ca.* Rickettsia spp. (Gottlieb et al., 2006; hereafter, *Cardinium*, *Wolbachia,* and *Rickettsia*, respectively), have been reported at low frequencies (Himler et al., 2011). These types of endosymbionts manipulate reproduction to their own advantage in a number of arthropods (Engelstädter & Hurst, 2009) and may spread rapidly through a population once introduced (Werren, Baldo, & Clark, 2008). For example, cytoplasmic incompatibility (CI) results in death of offspring produced by females uninfected with *Wolbachia*, when eggs are fertilized by a male‐symbiont carrier (Werren et al., 2008). The species *Ca.* Fritschea bemisiae (Everett, Thao, & Horn, 2005) and *Ca.* Hemipteriphilus asiaticus (Bing et al., 2013) have also been reported from whiteflies, although they have been relatively unstudied.

Host shifting of endosymbionts, also referred to as horizontal transmission (or transfer), can influence the spatio‐temporal dynamics in whitefly mitotype diversity and population structure (Caballero, 2007; Himler et al., 2011; Hurst & Jiggins, 2005) by favoring the establishment or displacement of one mitotype over another, particularly when conferring adaptive traits (Feldhaar, 2011; Himler et al., 2011). Environment‐associated niche adaptation of *B. tabaci* can be influenced by endosymbiont community composition, which in turn can alter the distribution of different vector mitotype–virus combinations. In Pakistan and India, studies have shown that *B. tabaci* mitotypes native to Asia that transmit cotton leaf curl disease‐begomoviruses (CLCuD; Bedford et al., 1994) have undergone geographic expansion and/or contraction, while at the same time the composition of the cotton leaf curl disease complex has been in flux, potentially corresponding to mitotype fluctuations (Ahmed et al., 2011; Ashfaq et al., 2014; Paredes‐Montero et al., 2019). Although many studies have characterized a number of different begomoviral species and strains associated with CLCuD, only a few have considered whitefly vector–endosymbiont relationships and their dynamics in an ecological context. Although precedents exist for host shifting among secondary endosymbionts (Baumann, 2005; Russell, Latorre, & Sabater‐Munoz, 2003), there are no reports of such shifting among obligate endosymbionts of *B. tabaci*. However, under conditions of ecological overlap, the potential for horizontal transfer of endosymbionts cannot be ruled out (Clark et al., 2000).

The objectives of this study were to determine the composition and rate of fixation of bacterial primary and secondary endosymbionts in relation to the spatial distribution of *B. tabaci* mitotypes in the Punjab region of Pakistan. Mitotypes were identified using the mitochondrial cytochrome oxidase I gene (mt*COI*) sequence as a molecular marker, whereas the composition and frequency of bacterial operational taxonomic units (OTUs) were based on ribosomal 16S RNA gene (rDNA) sequence comparisons. Also, the potential for horizontal transmission of secondary symbionts between the endemic mitotypes of *B. tabaci* was investigated by PCR amplification, sequencing, and phylogenetic analysis of the 16S rRNA gene sequence.

## 2 MATERIALS AND METHODS

### 2.1 Whitefly samples, DNA isolation, and mitotype identification

Whitefly adults were collected from infested cotton, vegetables, and noncrop species from agricultural and urban landscapes representing eleven cotton‐growing districts in Pakistan, Bahawalnagar, Bahawalpur, Khanewal, Lodhran, Multan, Okara, Pakpattan, Rahim Yar Khan, Sahiwal, Shaheed Benazir Abad, Vehari, and in one noncotton‐growing district, Lahore (Table S1). Three or more whitefly samples were collected at an average distance of ~500 feet apart per collection site. A whitefly sample consisted of individuals from all infested leaves in a single plant that was collected using a hand‐held aspirator, with a minimum of six individuals per plant. Whiteflies were collected live into a 1.5‐ml microfuge tube containing 95% alcohol and stored at −20°C. Mitotypes that are week competitors are expected to become rare in the presence of the apparently more aggressive Asia II‐1. Such mitotypes, such as Asia II‐5 and II‐7, are presently only found in urban landscapes where their numbers are also limited (*n* = 3–9). This may be further explained by the minimal to no use of pesticides in the urban areas, which leaves the natural enemies relatively undisturbed, compared to agricultural areas where pesticide use during the cotton‐growing season is frequent to control the whitefly vector, with the caveat that its frequent use often leads to increases in whitefly population sizes (Anthony, Brown, Markham, & Ffrenchconstant, 1995; Bedford et al., 1994; Denholm, Cahill, Dennehy, & Horowitz, 1998; Horowitz & Ishaaya, 2014). This hypothesis is consistent with the observed small available sample sizes of the Asia II‐5 (*n* = 3) and II‐7 (*n* = 23) mitotypes (herein) and the results of previous studies reporting similarly low frequencies within Pakistan agroecosystems (Ahmed et al., 2011; Ashfaq et al., 2014; Masood et al., 2017; Paredes‐Montero et al., 2019).

Total DNA was isolated from 276 adult whiteflies. The single whitefly extractions were performed using the method described by Zhang, Uyemoto, and Kirkpatrick (1998) with the following modifications. Individual whiteflies were blotted on filter paper to absorb ethanol, macerated in 600 µl of CTAB buffer containing 100 mM Tris‐HCl pH 8.0, 20 mM EDTA pH 8.0, 1.4 M NaCl containing 0.2% 2‐mercaptoethanol, and 2% hexadecyltrimethylammonium bromide, and incubated at 65°C for 15 min. One volume (vol) of chloroform was added, and the contents were mixed. The emulsion was broken by centrifugation at 13,000 g at 4°C for 3 min. One vol isopropanol and 40 μg glycogen were added to the supernatant and incubated for 10 min at 4°C for 10 min. The pellet was collected by centrifugation at 12,000 rpm for 10 min at 4°C, washed with 70% ethanol, air‐dried, and dissolved in low TE buffer (10 mM TrisCl‐EDTA, pH 8.0).

The 3′‐mt*COI* gene fragment (~850 bases) (Frohlich, Torres‐Jerez, & Bedford, 1999) was amplified by polymerase chain reaction (PCR) using the primers, C1‐J‐2195 (5′‐TTGATTTTTTGGTCATCCAGAAGT) and L2‐N‐3014 (5′‐TCCAATGCACTAATC TGCCATATTA). Each reaction contained 1× Jumpstart REDTaq ReadyMix (Sigma‐Aldrich), primers (0.4 μM each), 20 ng whitefly DNA, and double‐distilled water to a final vol of 25 μl. The PCR parameters were as follows: initial denaturation at 95°C for 2 min, followed by 30 cycles of 95°C for 60 s, 52°C for 60 s, and 72°C for 60 s, with a final extension at 72°C for 10 min. The amplicons were visualized by agarose gel (1%) electrophoresis in 1× TAE buffer, pH 8.0, containing 1× gel red (Biotium) at 100 V for 50 min. Amplicons of expected size were cloned into the TA cloning vector pGEM‐T Easy (Promega) by heat shock‐mediated transformation of *E. coli* DH5α competent cells. The colony PCR amplification (Gussow & Clackson, 1989) was carried out using primers M13F (5'‐TGTAAAACGACGGCCAGT) and M13R (5'‐AGGAAACAGCTATGACCATG; Promega) to identify clones containing the expected size insert. Cycling conditions were an initial denaturation at 94°C for 10 min, 35 cycles at 94°C for 60 s, 53°C for 60 s, and 72°C for 1 min, and final extension at 72°C for 10 min. The cloned insert was sequenced bidirectionally using an ABI 3700 capillary sequencer at the Genomics Core Facility, The University of Arizona, Tucson, Arizona, USA (http://uagc.arizona.edu).

For mitotype identification, reference *B. tabaci* mt*COI* sequences archived in the JK Brown lab database (unpublished and published) were selected to represent the seven previously recognized major phylogeographic clades of *B. tabaci* (Brown et al., 2010). Sequences were aligned using MUSCLE v3.8.31 (Edgar, 2004) implemented in Mesquite 2.75 (Maddison & Maddison, 2018). Aligned sequences were trimmed to 725 bp (~coordinates 767–1463) to remove priming sites and misaligned ends. The terminal gaps were treated as missing data.

Potential nuclear insertions of mitochondria (NUMTS) were detected by scanning mt*COI* sequences for ambiguities, indels, singletons, and stop codons, using previously described methods (Song, Buhay, Whiting, & Crandall, 2008). Phylogenetic analysis of the whitefly mt*COI* sequences (*n* = 3 per collection) was carried out by the neighbor joining (NJ) method, implemented in Mega v6 (Tamura, Stecher, & Peterson, 2013). All sequences identified as outliers to *B. tabaci* or that shifted among the different clades following multiple test runs were removed. All of the sequences from the same whitefly that shared 100% nt identity were “collapsed” using FABOX v1.41 (http://users-birc.au.dk/biopv/php/fabox/) to create a single haplotype sequence, and of these, one haplotype per whitefly was subjected to NJ analysis.

The optimal model of evolution was determined using jmodeltest software v2.1.7 (Darriba, Taboada, Doallo, & Posada, 2012) based on a “majority rule” consensus of information criterion (AIC), Bayesian information criterion (BIC), corrected Akaike information criterion (AICc), and decision theory performance‐based selection (DT). The general time reversible model of evolution with invariable sites (GTR + I) (Lanave, Preparata, Sacone, & Serio, 1984) was determined as the best‐fitting model for phylogenetic analysis.

Bayesian phylogenetic inference (BI) was carried out with BEAST v 1.8.4 (Bouckaert et al., 2014). Four independent Markov chain Monte Carlo (MCMC) computations, consisting of four Markov chains each, were carried out for 1 × 10^9^ generations. Trees were sampled every 10,000 generations, and log‐likelihood scores were plotted against generations sampled using Tracer v 1.6 (Bouckaert et al., 2014) to verify the stability of convergence of the chain and determine “burn‐in.” Each run was monitored based on effective sample sizes (ESS) to confirm a threshold of ≥200 was achieved. The MCMC log and tree files were combined using the LogCombiner software v1.8.4 (Drummond & Rambaut, 2007). Trees from the first 1 × 10^8^ generations per replicate were discarded (burn‐in). The 50% majority rule consensus tree was constructed with TreeAnnotator v1.8.4 (Drummond & Rambaut, 2007) and drawn in FigTree v1.4.2 (http://tree.bio.ed.ac.uk/software/figtree/). The mt*COI* sequences were assigned to one of the seven major phylogeographic clades and a mitotype within (Figure S1).

### 2.2 Endosymbiont 16S rRNA gene amplification and sequencing

The *16S rDNA* (~1,500 bp) sequence was PCR‐amplified using “universal” primers 27F (5′‐AGAGTTTGATCMTGGCTCAG) and 1513R (5′‐ACGGYTACCTTGTTACGACTT; 0.4 μM; Weeks, Velten, & Stouthamer, 2003; Weisburg, Barns, Pelletier, & Lane, 1991). The PCRs were set as explained above using 20 ng of whitefly DNA. Cycling parameters were an initial denaturation at 94°C for 2 min, and 35 cycles of 94°C for 30 s, 55°C for 30 s, and 72°C for 2 min, with a final extension at 72°C for 10 min (This study). Amplicon sizes were verified by agarose gel electrophoresis and cloned. Twelve clones per whitefly were selected by colony PCR amplification and sequenced, as described. Because all *B. tabaci* were expected to harbor *Arsenophonus*, and several samples yielded no amplicon by PCR amplification with universal primers, they were subjected to PCR amplification of the *23S rDNA* sequence using *Arsenophonus*‐specific primers Ars23S‐*F* (5′‐CGTTTGATGAATTCATAGTCAAA) and Ars23S‐R (5′‐GGTCCTCCAGTTAGTGTTACCCAAC; Thao & Baumann, 2004) to PCR amplify an expected 600 base pair size amplicon. The positive control was total DNA purified from *B. tabaci* previously identified as Asia II‐1 known to harbor *Arsenophonus* based on previous analysis (authors unpublished). The PCR was set as described above using 20 ng of whitefly DNA. Cycling conditions were an initial denaturation at 95°C for 5 min, and 30 cycles of 95°C for 30 s, 60.5°C for 30 s, and 72°C for 45 s, with a final extension at 72°C for 10 min (Thao & Baumann, 2004). The size of amplicons was confirmed by agarose gel (1%) electrophoresis. Samples yielding a band of equal intensity to the positive‐*Arsenophonus* control were scored as positive.

The *16S rDNA* sequences (universal) were assembled using SeqMan Pro software available in DNASTAR Lasergene v8.0 (DNASTAR, Inc.). Reads and chromatograms were inspected for misalignment and manually edited to identify high‐quality contigs with no conflicts (0%) between forward and reverse reads. Sequences were exported in FASTA file format and aligned using the SILVA Incremental Aligner (SINA) v1.2.11, a ribosomal structure‐sensitive tool accessing more than two million bacterial reference sequences (Pruesse et al., 2007). Sequences were trimmed to 1,084 bp using Mesquite v 2.75 (Maddison & Maddison, 2018). Chimeras and sequence contaminants were removed using the *uchime* (Edgar, 2004 *), classify.seqs,* and *removed.lineage* algorithms, implemented in Mothur v1.43.0 (Schloss et al., 2009).

The *16S rDNA* sequences were annotated using the SILVA reference database v1.2.11 (http://www.arb-silva.de; Schloss et al., 2009). A database was built in which sample information was summarized, including the sample collection information and the results of whitefly mitotype and endosymbiont analysis (Table S1). The relative frequency of each endosymbiont per mitotype by collection site was calculated using *barplot* using the graphics v3.6.0 package available in R (R Core Team, 2018).

### 2.3 Bacterial operational taxonomic unit identification and diversity

A *16S rDNA*sequence was designated as an “OTU” based on the a priori‐established cutoff of ~3% dissimilarity (Stackebrandt & Goebel, 1994) using the *get.dist* and *cluster* algorithms in Mothur v1.43.0 (Schloss et al., 2009). The relative frequencies of OTUs per mitotype were summarized in a contingency table with the *make.share* algorithm (Table S2) in Mothur v1.43.0. The OTUs were classified into taxa using the *classify.seqs* algorithm available in the SILVA taxonomy reference database v1.2.11 to calculate the probability of a query belonging to a taxonomic group in the reference dataset (Schloss et al., 2009). The relative frequency of bacterial OTU classes and families per mitotype was calculated, and frequencies were summarized using the *barplot* function in the graphics R package v3.6.0 (Core Team, 2018). To determine whether sampling was statistically sufficient to portray the majority of whitefly OTU richness, rarefaction curves were calculated for 1,000 randomized runs per whitefly using the *rarefaction.single* command in Muther v1.43.0 (Schloss et al., 2009). A contingency table containing OTU genus assignments was produced in Mothur, and the OTU relative abundance per whitefly mitotype was depicted by a heatmap. A dendrogram that grouped the heatmap columns (mitotype assemblage) by relatedness was constructed using a Euclidean distance matrix, and a Pearson correlation coefficient test (Pearson, 1895) was carried out to analyze the robustness of the groupings. The heatmap and dendrogram were built in R packages *Gplots* (Warnes et al., 2019) and *Hmisc* (Harrell, 2018), respectively.

The extent of endosymbiont diversity within and between whitefly host mitotype was determined based on alpha and beta diversity indices using the *summary.single* and *summary.shared* functions, respectively, in Mothur v1.43.0. The nonparametric estimator Chao1 (Chao, 1984), Shannon index (*H*) (Shannon, 1948) that assumes random sampling of OTUs for each mitotype group, and the Simpson index (*D*) (Simpson, 1949), which assigns weights to OTUs based on abundance, were selected as the alpha diversity indicators. The beta diversity indices estimated were the Jaccard index (Jaccard, 1912) to compare between‐mitotype shared and distinct OTUs, the Sorensen index (Sørensen, 1948) that assigns a low weight to singletons, compared to commonly occurring OTUs, and Yue and Clayton (Thetayc) index (Yue & Clayton, 2005) that evaluates dissimilarity of OTU assemblages between mitotypes, based on abundance of each OTU. The differences between mitotypes were assessed for significance using a 95% confidence interval and 1,000 bootstrap iterations.

### 2.4 Arsenophonus endosymbiont phylogeny

The *Arsenophonus* 16S phylogeny was reconstructed by Bayesian analysis using a single representative sequence per *Arsenophonus* OTU and nonredundant haplotypes. The tree was rooted with the *Hamiltonella 16S rDNA*, the primary endosymbiont of the pea aphid, *Acyrthosiphon pisum* (Harris). Selected reference sequences were *B. tabaci*‐*Arsenophonus* exemplars of the *B. tabaci* major clades, Asia I, Asia II, and Sub‐Saharan Africa. Sequences for *B. tabaci* and non‐*B. tabaci* were obtained from the JK Brown lab database or the GenBank database. The *Arsenophonus* outgroups selected were endosymbionts of the whiteflies, *Aleurodicus dispersus* (Russell), *A. dugessi* (Cockerell), *Tetraleurodes acaciae* (Quaintance), and *Trialeurodes vaporariorum* (West). The best‐fitting model was Tamura–Nei with gamma distribution rate variation and TrN + G+I (invariable sites). Bayesian analysis was carried out as described above.

## 3 RESULTS

### 3.1 Whitefly *B. tabaci* mitotypes

**Figure 1:**
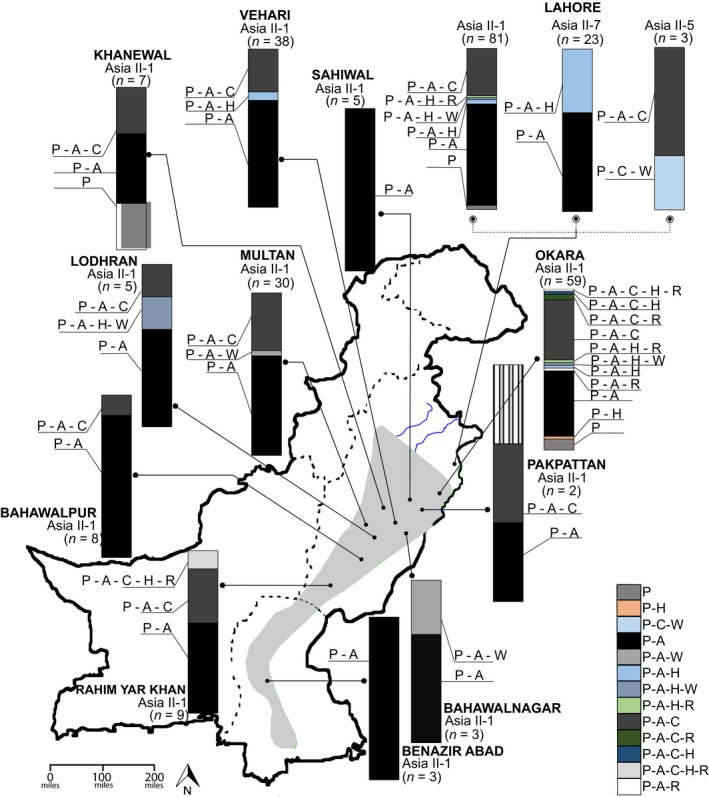
Distribution of endosymbiont assemblages in 12 districts of Pakistan. Bars represent the relative abundance of all possible combinations of the six common genera reported in *Bemisia tabaci*, including the primary *Portiera* and five secondary endosymbionts. *B. tabaci* mitotypes Asia II‐5 and II‐7 were only recorded from Lahore, whereas Asia II‐1 is widely distributed. Capital letters denote the species as follows: *Portiera* (P), *Arsenophonus* (A), *Cardinium* (C), *Wolbachia* (W), *Rickettsia* (R), and *Hemipteriphilus* (H), where N equals the number of whitefly samples. Blue lines represent rivers, dotted lines indicate provincial limits, and polygon shows the cotton‐growing area in Pakistan

The Bayesian analysis of mt*COI* sequences for whitefly *B. tabaci* field collections indicated they represented mitotypes that grouped in the major clade Asia II (Brown et al., 2010) as three well‐supported subclades (>95% pp): Asia II‐1, II‐5, and II‐7 (Dinsdale, Cook, & Riginos, 2010). Asia II‐1 was the predominant mitotype identified in the cotton agroecosystem, at 90.6% (*n* = 250) relative frequency. The second most prevalent was Asia II‐7 occurring in collections from Lahore (urban area), at 8.3% (*n* = 23) frequency. The Asia II‐5 mitotype occurred at the lowest frequency among the three mitotypes identified, at 1.1% (*n* = 3), making it the least represented mitotype in Punjab province (Figures 1 and S1). To reduce repetitiveness, the term “relative frequency,” which is based on the percentage of the total number of samples, hereafter, is indicated as “frequency.”

### 3.2 Endosymbiont OTU identification and characterization

The bacterial endosymbionts represented 43 bacterial genera in the nine classes: Acidimicrobiia, Actinobacteria, Alphaproteobacteria, Bacilli, Bacteroidia, Betaproteobacteria, Gammaproteobacteria, Rubrobacteria, and Sphingobacteriia (Table S3). All of the bacterial classes were associated with Asia II‐1. By comparison, the Actinobacteria, Alphaproteobacteria, Bacilli, Betaproteobacteria, and Gammaproteobacteria were identified in Asia II‐7 mitotypes, while the Asia II‐5 mitotypes harbored only three classes, Alphaproteobacteria, Bacteroidia, and Gammaproteobacteria. Among the nine bacterial classes, only Alphaproteobacteria and Gammaproteobacteria were found in all three of the Asia II mitotypes.

The most prevalent families were as follows: *Bacteroidaceae, Enterobacteriaceae,* and *Halomonadaceae*, representing ~90% of the *16S rDNA* sequences across the three mitotypes. The remaining 10% were detected only in the Asia II‐1 mitotype, which represented six bacterial classes and thirty families (Table S3). These diverse bacteria appear to represent an assortment of previously uncharacterized bacterial gut flora and/or extraneous plant endophytes.

Among the most prevalent genera (Tables S1 and S2) were those previously recognized as endosymbionts of *B. tabaci*. These were *Portiera* (Gammaproteobacteria), the obligate primary endosymbiont, *Arsenophonus* (Alphaproteobacteria), one of two known mitotype‐specific major secondary endosymbionts, and *Cardinium* (Bacteroidia), a minor secondary endosymbiont known to occur in many other insects (arthropods), including *B. tabaci*.

The endosymbionts *Portiera* and *Arsenophonus* occurred at a frequency of 100% and (Figure S2a) and 93% (Figure S2b), respectively, among *B. tabaci* from the twelve districts sampled in Pakistan, regardless of the particular *B. tabaci* mitotype. Among the secondary endosymbionts previously reported from *B. tabaci*, only *Fritschea* and *Hamiltonella* were not detected among the *B. tabaci* samples analyzed in this study. The *Cardinium* (Bacteroidia) was the second most abundant secondary endosymbiont. Though it was commonly associated with Asia II‐1 and Asia II‐5 mitotypes, it was conspicuously absent (undetectable) from the Asia II‐7 (Figure S2c). In 7% of the Asia II‐1 samples from the districts of Khanewal, Lahore, and Okara (Figure S2b) and one Asia II‐5 sample from Lahore, *Arsenophonus* was not detected by PCR amplification of the 16S or the 23S rRNA genes.

Among the other secondary endosymbionts identified, *Cardinium* was detected in 29% of *B. tabaci* samples, while 8% harbored *Hemipteriphilus* (Figure S2d), and 4% carried either *Rickettsia* (Figure S2e) or *Wolbachia* (Figure S2f). *Cardinium* and *Wolbachia* were detected in Asia II‐1 and II‐5, but absent in Asia II‐7. For the Asia II‐1 samples (*n* = 192) from Lahore, Lodhran, Okara, Rahim Yar Khan, and Vehari, *Hemipteriphilus* occurred at a frequency ranging from 5% to 20%, while infection of Asia II‐7 (*n* = 23) occurred at a frequency of 39%, while it was not detected at all the Asia II‐5 mitotype. Finally, *Rickettsia* was identified in the Asia II‐1 mitotype only and at a frequency of 2% in the individuals from Lahore, Okara, and Rahim Yar Khan (Figure S2e).

One of thirteen combinations of bacterial genera or “assemblages” existed as co‐infections among all whiteflies sampled. Among them, the *Portiera*–*Arsenophonus* (P‐A) and *Portiera*–*Arsenophonus*–*Cardinium* (P‐A‐C) assemblages were most prevalent, at a 57% and 27% frequency, respectively. The assemblage consisting of P‐A predominated in whiteflies from the Sahiwal and Okara districts and were present in all three mitotypes at a frequency of 100% and 40%, respectively, and to lesser extents in all mitotypes from the other cotton‐growing districts of Punjab province (Figure 1). In the Sindh province (Figure 1), where the Asia II‐1 mitotype has recently undergone genetic and population expansions (Ashfaq et al., 2014; Paredes‐Montero et al., 2019), only the P‐A assemblage was detected. By comparison, the P‐A‐C assemblage occurred in both the Asia II‐1 and II‐5 mitotype in nine of the twelve districts, respectively, while the Asia II‐7 mitotype (*n* = 23) harbored P‐A and P‐A‐*Hemipteriphilus* (P‐A‐H) assemblages at a frequency of 61 and 39%, respectively. Despite an extensive number of samples tested (*n* = 250), the P‐A‐H assemblage was detected in only 2% of Asia II‐1 samples. The P‐A‐H together with either *Cardinium* (P‐A‐H‐C), *Rickettsia* (P‐A‐H‐R), *Wolbachia* (P‐A‐H‐W), or *Cardinium* with *Rickettsia* (P‐A‐H‐C‐R) were detected at a frequency of 0.4, 0.8, 1.2, and 0.8%, respectively. The P‐C‐*Wolbachia* (P‐C‐W) assemblage occurred in Asia II‐5 at a frequency of 33%, while the Asia II‐1 mitotype from Okara district harbored P‐A‐*Rickettsia* (P‐A‐R), at a frequency of only 3% (Figure 1).

### 3.3 Bacterial operational taxonomic unit identification, diversity, and structure

**Figure 2:**
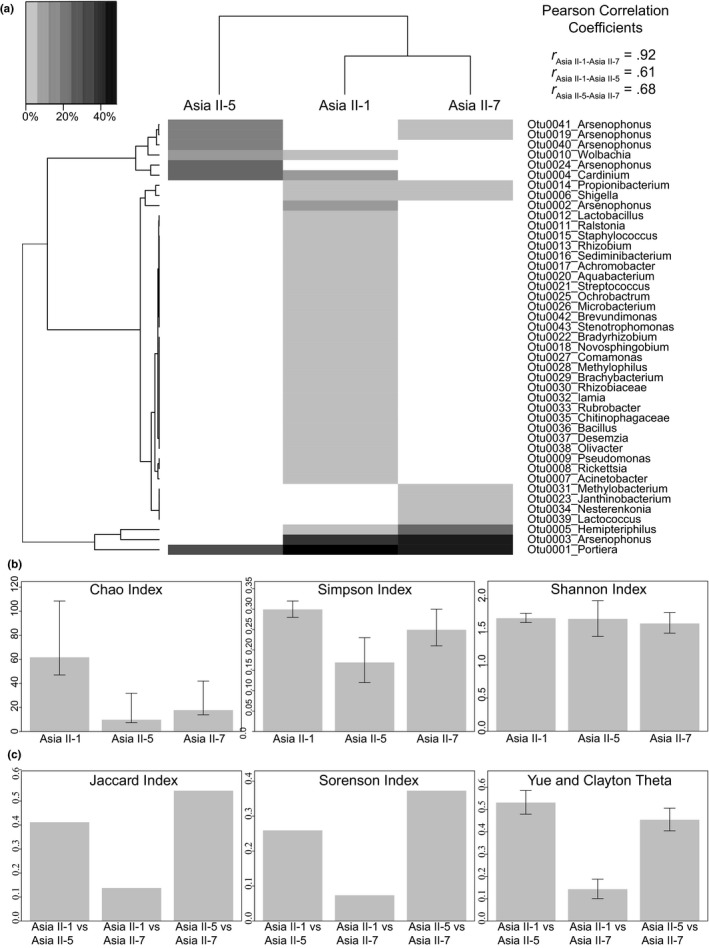
Heatmap of relative abundance of 43 operational taxonomic units (OTUs) clustered using 1,084 bp the 16S ribosomal RNA gene (*16S rRNA*). Dendrogram shows Euclidean correlation among OTUs (vertical) and mitotypes (horizontal) based on the relative abundance and shared OTUs, respectively (a). The clustering of Asia II‐1 and Asia II‐7 subclades is supported by a Pearson correlation coefficient of .92 (*p* < .05). Diversity indicators (b) alpha and (c) beta for OTUs associated with the three *Bemisia tabaci* mitotypes identified in this study. Bars correspond to the 95% confidence intervals

The rarefaction curves for *16S rDNA* sequences were not asymptotic among the three mitotypes and were taken as an indication that OTU diversity sampling was not exhaustive (Figure S3). Based on an OTU “species” threshold of 3% pairwise divergence, 43 OTU groups were recovered. The relative abundance of OTUs associated with all three mitotypes (Figure 2a, Tables S2 and S4) was consistent with results of a previous study involving six of the seven known phylogeographic clades (mitotypes) of *B. tabaci* (Zchori‐Fein & Brown, 2002). Hierarchical clustering of mitotypes by relative OTU abundance indicated that Asia II‐1 and Asia II‐7 mitotypes harbored many of the same OTUs, while many of the OTUs harbored by the Asia‐5 mitotype were unique (Figure 2a; Pearson's correlation coefficient 0.92, *p* < .05; Tables S2 and S4). Among the 43 unique OTUs, Asia II‐1 harbored 63%, while 10% and 4% were associated with Asia II‐7 and Asia II‐5, respectively.

Alpha diversity (Figure 2b) calculators showed that OTU diversity was the greatest for the Asia II‐1 mitotype, followed by II‐5 and II‐7, respectively. The Chao index (Figure 2b) for Asia II‐1, at 61.86, was higher than Asia II‐5 and II‐7, at 10 and 18, respectively. The Chao 1 confidence intervals predicted statistically significant differences in diversity between Asia II‐1 and the other two mitotypes. Although the Simpson index also predicted that Asia II‐1 harbored the greatest diversity, followed by Asia II‐7, the result was not statistically supported (Figure 2b). The Shannon index predicted statistically similar richness among the three mitotypes that ranged from 1.63 to 1.71; however, the statistical support was low (Figure 2b) indicating that assumptions for random sampling and equal representation of OTUs among mitotypes were not met (Shannon, 1948).

The Jaccard, Sorenson, and Yue–Clayton theta indices, at 0.14, 0.07, and 0.14, respectively, predicted the least dissimilarity between Asia II‐1 and II‐7, with high statistical support (Figure 2c), whereas a distant relationship was predicted between the Asia II‐5 mitotype and Asia II‐1 and II‐7. This observation was consistent with Pearson's correlation coefficient of .92 (*p* < .05) for the dendrogram favoring a close relationship of Asia II‐1 and II‐7, with Asia II‐5 being an outlier (Figure 2a). In general, diversity indices revealed a dependency on microbiome composition and biological relationships among and between mitotypes.

### 3.4 Portiera 16S rDNA OTU analysis

The nucleotide pairwise distances calculated for all *Portiera* sequences combined and based on a 3% threshold were extremely narrow, ranging from 0% to 0.67% divergence and effectively identifying all *Portiera* sequences as a single OTU (Table S2). The same pattern was observed for *Cardinium*, *Hemipteriphilus*, *Rickettsia*, and *Wolbachia* sequences, with intra‐OTU pairwise divergences below 3% nt dissimilarity (Figure 2a).

### 3.5 Arsenophonus 16S rDNA OTU phylogeny

**Figure 3:**
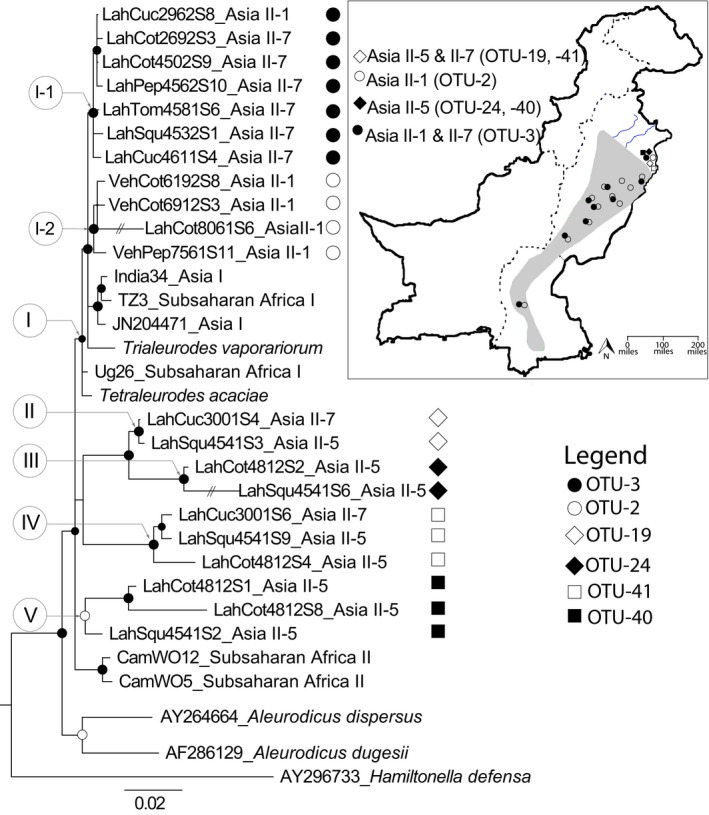
Phylogeny of *Arsenophonus* based on the 16S ribosomal RNA gene (*16S rRNA*, 1,084 bp), for *Bemisia tabaci* mitotypes and other whitefly species. The tree was rooted using the *Hamiltonella 16S rDNA* sequence from the aphid *Acyrthosiphon pisum.* Closed and opened circles at nodes depict posterior probabilities above 95% and 80%–94%, respectively. The Roman numerals I‒V represent well‐supported clades of *Arsenophonus* associated with the three mitotypes identified in Pakistan, Asia II‐1, ‐5, and ‐7. Subclade I‐1 indicates the *Arsenophonus* operational taxonomic unit (OTU) associated with both Asia II‐1 and II‐7, whereas subclade I‐2 shows OTU uniquely associated with Asia II‐1. Closed circles at the tree tip depict representative sequences of OTU‐003, detected in Asia II‐7 and II‐1. Open circles show sequences that group with OTU‐0002, uniquely found in Asia II‐1. Closed diamonds show the OTU‐0024, and closed squares indicate OTU‐0040, both uniquely associated with Asia II‐5. Opened diamonds depict OTU‐0019, and opened squares depict OTU‐0041, uniquely detected in Asia II‐5 and II‐7. The map shows OTU distribution by *B. tabaci* mitotype, the blue lines represent rivers, the dotted lines indicate provincial limits, and the polygon shows the cotton‐growing areas of Pakistan

Phylogenetic analysis of the *16S rDNA* resolved five *Arsenophonus* clades (I‒V). The tree structure for clades I through V (Figure 3) was consistent with OTU groups with 3% or greater nt divergence (Figure 2a). The two well‐supported subclades within clade I, herein, I‐1, and I‐2 contained the OTU‐003 and OTU‐002, which were reminiscent of previously reported for strains AI2 and AI1 (Singh et al., 2012), respectively. The OTU‐003 was most widespread, occurring in the Asia II‐1 and II‐7 mitotypes. The OTU‐002 (clade I‐2, Figure 3) was nested within clade I, and occurred uniquely in Asia II‐1, the most widespread mitotype in Pakistan.

Clade I contained the same *Arsenophonus* OTUs also identified among *B. tabaci* mitotypes represented by three other major clades, Asia I, II, and Sub‐Saharan Africa as well as two other whiteflies *T. acaciae* and *T. vaporariorum* (Figure 3). This pattern was also observed for the *Cardinium* and *Wolbachia* OTU in the Asia II‐1 and II‐5 mitotypes, and the *Hemipteriphilus* OTU harbored by the Asia II‐1 and II‐7 (Figure 2a). The presence of the same OTUs in multiple mitotypes of *B. tabaci* and/or two non‐*Bemisia* whiteflies was suggestive of interspecific horizontal transfer of the OTU (Thao & Baumann, 2004).

The *Arsenophonus* OTUs 019 and 041 grouped in clades II and IV, respectively, and occurred in Asia II‐5 (*n* = 2) and II‐7 (*n* = 2). In contrast, *Arsenophonus* OTUs 024 and 040 were rare, grouped in clades III and V, respectively, and occurred only in Asia II‐5 mitotypes (*n* = 2) from Lahore. In both instances, they diverged from the most abundant *Arsenophonus* species by 3%–7% (Figure 3). By comparison, *Arsenophonus* OTUs associated with *B. tabaci* from Cameroon, “CamWO5” and “CamWO12,” members of the Sub‐Saharan Africa‐West subclade, diverged by 3%–5% from OTUs in the other *Arsenophonus* clades (Figure 3). The *Arsenophonus*‐*A. dispersus* (GenBank Accession no. AY264664) and *A. dugessi* (Accession no. AF286129) [subfamily Aleurodicinae] grouped in the basal position of the tree and diverged by 3%–8% from *Arsenophonus* OTUs associated with the Aleyrodinae (subfamily), perhaps indicative of subfamily specific OTU barriers (Clark et al., 2000). The results together support a pattern of OTU environmentally related contributions among whiteflies in general, including different mitotypes of *B. tabaci*.

## 4 DISCUSSION

The whitefly *B. tabaci* collected in the Punjab province of Pakistan were identified as mitotypes Asia II‐1, ‐5, and ‐7 (Dinsdale et al., 2010), which belong to the Asia II major clade of *B. tabaci* (Brown et al., 2010; de Moya et al., 2019). Characterization of the endosymbiont communities associated with endemic *B. tabaci*, with respect to composition, distribution, and ecological microniche(s) within of the Punjab district, suggests that certain mitotype–endosymbiont communities may have been/be selectively associated with previous and current outbreaks of cotton leaf curl disease, respectively. The leaf curl disease is caused by approximately ten endemic, divergent begomoviral species, and strains (begomovirome) that cycle in cultivated and wild plant hosts in the agroecosystem, as single infections or as mixtures from which viral recombinants emerge and spread throughout the region (Zubair et al., 2017). Based on complex multitrophic interactions involving different *plant host–virome–mitotype vector* combinations, the endosymbiont community is hypothesized to contribute to niche‐specific adaptation of *B. tabaci* mitotypes in the urban‐ and agroecosystems, the latter pronouncedly influenced by cropping and pesticide use regimes (see references in Bedford et al., 1994; Brown & Czosnek, 2002). At the same time, widespread planting of genetically uniform‐cotton genotypes, with differential virome‐resistance, selects for most resilient begomovirome that together with the most‐fit mitotype, and the optimal mitotype–virome transmission competency, dictate the composition and prevalence of the viral pathogen and whitefly vector counterpart in the agroecosystem at any given time. Such scenarios, though still speculative, appear to have been playing out in the Punjab province for nearly thirty years, since the first leaf curl disease outbreak that led to CLCuMV epidemic in cotton beginning in ~1990 (Mansoor et al., 2003).

The results of bacterial profile characterization for the three Asia II mitotypes showed that among the 43 OTUs identified for all mitotypes combined, the OTU distribution differed for each mitotype. The composition of OTU assemblages associated with different mitotypes can serve as useful indicators of the genetic/genomic relatedness among the three Asia II mitotypes and so is seen as an informative tool together with whitefly mitotype determination for investigating large‐scale relationships among global mitotypes.

The obligate, primary endosymbiont, *Portiera* OTU was detected 100% of the time in the three mitotype‐associated 16S profiles. In contrast, the secondary *Arsenophonus* endosymbiont OTUs were shown to occur at relative frequencies of 93% based on 16S and 23S rRNA gene amplification. This result was expected because there is no instance in which *B. tabaci* has been found to lack a facultative secondary endosymbiont, either *Arsenophonus* or *Hamiltonella* (Gueguen et al., 2010; Zchori‐Fein & Brown, 2002).

Other endosymbionts previously shown to disrupt reproductive patterns of their arthropod host, including *Cardinium*, also infect *B. tabaci*. This bacterium has been shown to alter the sex ratio and lead to rapidly increased population size due to a greater number of female offspring. Greater population sizes of *B. tabaci* in turn can readily accelerate begomovirus transmission rates and cause disastrous outbreaks that become epidemic in a very short period of time. The endosymbiont, *Cardinium,* was detected in 29% of the *B. tabaci* mitotypes analyzed in this study, compared to other endosymbionts also known to influence reproductive outcomes, *Rickettsia*, and *Wolbachia*,which were minor components of the community, with detection in 8%, and 2% of samples, respectively.

### 4.1 Arsenophonus 16S rDNA phylogeny

A comparison of the phylogenetic tree of the secondary endosymbiont *Arsenophonus* (Figure 3) with that of its *B. tabaci* host tree (Figure S1) showed a pattern reminiscent of horizontal transmission, in which heritable facultative bacterial endosymbionts have been transferred either between related or divergent insects (Gonella et al., 2015; Gueguen et al., 2010; Mansoor et al., 2003; Oliver, Degnan, Burke, & Moran, 2010; Thao et al., 2000; Tsuchida, 2004; Weiss et al., 2006). Such pattern is consistent with the niche overlap observed for the three mitotypes in the Lahore collection sites (Figure 3).

Horizontal transmission (transfer) of this symbiont is not exclusive to *B. tabaci* and has been previously identified among whiteflies in the subfamily, Aleyrodinae (Thao & Baumann, 2004). The evidence for “horizontal transfer” is based on the two rare *Arsenophonus* “putative species,” the OTU‐019 and OTU‐041, which are present in Asia II‐5 and II‐7 mitotypes, and on another unique “putative species,” OTU‐003, detected uniquely in Asia II‐1 and II‐7 mitotypes. A pattern reminiscent of horizontal transfer was also observed for the endosymbionts *Cardinium* and *Wolbachia* detected in both Asia II‐1 and II‐5 mitotypes and *Hemipteriphilus* detected in Asia II‐1 and II‐7. Intermitotype transfer of these OTUs can be envisioned based on documentation that the *B. tabaci* mitotypes were found in intimate proximity to one another at the Lahore sampling sites. The more restricted distribution of Asia II‐5 and II‐7 seems to coincide with their preference for the subtropical conditions in Lahore, while the Asia II‐1 mitotype favors the hot, arid environments of the southern Punjab province.

### 4.2 Whitefly co‐infection by endosymbionts

Studies of endosymbiont‐*B. tabaci* mitotype dynamics have provided insights into population dynamics, geographic distribution, and genetic/genomic structure of the *B. tabaci* group, a group as a whole for which most biological, genetic, and population‐level features are poorly understood (Himler et al., 2011; Tsuchida, 2004). Nineteen endosymbiont assemblages have been previously associated with global *B. tabaci* populations (see references in Zchori‐Fein et al., 2014). Among them, six have been confirmed for all three Asia II mitotypes in Pakistan, with six more identified uniquely in Asia II‐1 and ‐7 mitotypes, as well as *Hemipteriphilus* and *Cardinium*. In some instances, the composition and distribution of different bacterial assemblages in the context of biological (field) and evolutionary (phylogenetic) considerations are relatively informative. Based on endosymbiont composition, the phylogenetically confounded Asia II‐1 and Asia II‐7 “species” (Paredes‐Montero et al., 2019; Figure 2a) [“subclades”] were found to be more closely related to one another than to Asia II‐5. This conclusion is supported by the diversity estimates (mt*COI*) and the number and type of shared OTUs (Figure 2a,b). Based on insights gained from this dual‐molecular marker approach, an expanded study of endosymbiont composition–mitotype associations globally could aid greatly in addressing the extent of gene flow and delimitation of boundaries for the *B. tabaci* group.

The distribution of the three Asia II mitotypes of *B. tabaci* and their secondary endosymbiont assemblages appeared to have been influenced by the differences in climatic and ecological conditions at the different collection sites in Pakistan. The mitotype harboring the greatest number of bacterial assemblages was Asia II‐1 from Okara (humid area), with 12 of the 13 bacterial groups having been documented. This was followed by the Lahore whitefly samples that harbored six of the 13 groups. The climate microniches in Okara and Lahore are humid and subtropical, while by comparison, the other districts are characterized as either an arid desert or semi‐arid (Peel, Finlayson, & McMahon, 2007). The rare P‐A‐C‐H‐R assemblage was detected in only a small number of Asia II‐1 individuals, occurring in the Okara and Rahim Yar Khan districts where extreme summer temperatures are characteristic.

### 4.3 Endosymbiont prevalence

#### 4.3.1 Arsenophonus OTUs

A high rate of fixation was observed for the secondary endosymbiont *Arsenophonus* in the Asia II‐1 mitotype, at a frequency of 93%. In laboratory studies, the association of *Arsenophonus* populations of Asia II‐1 in India has been linked to reduced fitness, based on a study in which the endosymbiont was nearly eliminated by an antibiotic treatment. The “cured” whitefly showed increased fitness based on the production of more fit offspring, that is increased fecundity, as well as increased immature developmental time, higher survival rate, and longer lifespan (Raina et al., 2015).

Of interest, among the distribution patterns reported herein are that spread of *Arsenophonus* OTU‐002 and ‐003 (clades I‐1, ‐2, Figure 3) in *B. tabaci* Asia II‐1 in Punjab province occurred coincidently with a round of genetic expansion in the population, followed by a genetic bottleneck and “recovery” evident by a gradual increase in diversity (Paredes‐Montero et al., 2019). In this scenario, genetic expansion might be explained by an upsurgence of Asia II‐1 that followed an increase in the number of insecticide applications that apparently lead to the development of insecticide resistance (Ahmad, Arif, Ahmad, & Denholm, 2002; Ahmad & Khan, 2017). This hypothetical outcome is supported by documentation of the putative “newly expanded” OTU‐002 and ‐003 that was found widely distributed among *B. tabaci* mitotypes extant in the Punjab province and by the recent expansion of Asia II‐1 into the Sindh province from the north (Ashfaq et al., 2014). Although speculative, the patterns are suggestive of a possible relationship between Asia II‐1 individuals that harbor the *Arsenophonus* OTU‐002 and/or ‐003 and the second leaf curl epidemic caused by CLCuKoV‐Bur, extant in the vicinity of the leaf curl outbreak occurring during 2001–2004, after the recombinant had displaced CLCuMuV, the causal species of the 1990 outbreak, in the Punjab province. Routine surveillance would aid greatly in determining whether specific endosymbiont assemblages contribute to increased whitefly host fitness that encourages the expansion of aggressive/more fit *B. tabaci* mitotypes over others (contraction) and thereby alter the dynamics of virome composition due to mitotype‐specific transmission competency.

The results of pairwise divergence estimates (3% species threshold) and phylogenetic analyses (Bayesian and maximum likelihood) indicated all three Asia II mitotypes harbored six distinct *Arsenophonus* OTUs (Figure 3). Among them, Asia II‐1 uniquely harbored OTU‐002, while OTU‐003 occurred only in Asia II‐1 and II‐7. And, OTUs 024 and 040 were associated only with Asia II‐5, whereas Asia II‐5 and II‐7 uniquely harbored OTUs 19 and 041. These consistent differences in *Arsenophonus* diversity among the three mitotypes suggest that through an association with one or more homogeneous OTUs, fitness or other phenotypes can be influenced by endosymbiont‐mediated adaptation. This phenomenon is borne out by the observation that geographic distributions overlap with specific agricultural and/or urban environments in which samples were collected.

Several *Arsenophonus* OTUs have been reported from Asia II samples in India (Singh et al., 2012), Q‐eastern clade mitotypes in Israel, “Ms mitotype” endemic to Uganda and Reunion Island in the Indian Ocean, the presumed southward limit of its range (Gueguen et al., 2010; Zchori‐Fein et al., 2014). Studies have documented between four to five *Arsenophonus* OTUs associated with *B. tabaci* (Gueguen et al., 2010; Hashmi, Devi, Meshram, & Prasad, 2018; Kanakala & Ghanim, 2019). However, whether these OTUs are “species” of *Arsenophonus* or represent multiple copies of the same *16S rRNA* gene in the same individual requires additional investigation. Nonetheless, it is of interest that despite a large number of libraries were screened for Asia II‐1, only two OTUs were found associated with this mitotype, compared to the three and four *Arsenophonus* OTUs detected in Asia II‐7 and ‐5, respectively, adding to the hypothesis that diverging mitotypes of *B. tabaci* may harbor different *Arsenophonus* strains.

The multitrophic interactions involving host plant‐whitefly mitotype–endosymbiont assemblages–begomovirome reflect a great potential of ongoing evolutionary processes with measurable outcomes and potentially important short‐term consequences. Such complex interactions are not limited to the Asia II mitotypes, which are also endemic to China, India, and Pakistan, but rather extend throughout the entire *B. tabaci* group. As a putative cryptic species group, *B. tabaci* has long been known to exhibit phenotypic plasticity and an inherent capacity for adaptation to diverse climatic niches throughout the world, also locations where begomoviruses are diverse and genetically readily adaptive (Brown et al., 2010; Mound, 1963; Oliver et al., 2015). Based on the co‐adaptation of both partners in the same environment (host, climatic niche, other), a number of examples have been identified for which virus‐vector co‐evolutionary relationships have formed the basis for the preferential acquisition or “transmission competency” of certain begomoviruses over others (Brown et al., 2010; Brown & Czosnek, 2002).

### 4.4 Other secondary endosymbiont OTUs

The OTU identified as *Cardinium* occurred in Asia II‐1 and Asia II‐5 only. Due to an insufficient sampling of OTUs suggested by the rarefaction curves (Figure S3), *Cardinium* may occur at a frequency higher than the 29% reported here. The limitation of sample size (availability) for certain mitotypes may have resulted in biased estimated frequencies of detectable (or undetectable) OTUs. In particular, *Cardinium*, identified from a number of mitotypes worldwide, may occur at low, undetectable levels, and if so, studying a larger population size may show it also occurs in Asia II‐1 or II‐5 mitotypes. Infection of the polyphagous A mitotype of *B. tabaci* by *Cardinium* has previously been associated with increased fecundity (Caballero, 2007) and more female offspring, compared to lower fecundity of monophagous Jatropha mitotype that harbored *Wolbachia*. Thus, the eventual fixation of *Cardinium* in Asia II‐1 in Pakistan could also influence whitefly fitness and/or abundance, in turn influencing CLCuD epidemiology by increasing virus transmission rates due to high whitefly pressure. Indeed, increased fitness of Asia II‐1 has been previously reported in Pakistan and has been associated with shifts in population structure of Asia II‐1 in cotton fields (Ashfaq et al., 2014; Masood et al., 2017; Paredes‐Montero et al., 2019). Finally, based on an ability to perturb whitefly reproduction, the low (2%) frequency of *Wolbachia* in Asia II‐1 may already provide an important clue that it has in the past contributed to a previous expansion after which it was removed from much of the whitefly population.

Analysis of OTUs indicated the occurrence of *Rickettsia* in Asia II‐1 at a 2% frequency (Table S2, Figure S2). In Israel and the USA, where the B mitotype is endemic and introduced, respectively, *Rickettsia* has been reported at frequencies as high as 95%. In Israel, the B mitotype harboring *Rickettsia* was shown to be more susceptible to certain insecticides (Ghanim & Kontsedalov, 2009; Kontsedalov et al., 2008) and exhibit thermotolerance (Brumin et al., 2011). In contrast, the B mitotype, which was introduced in the southwestern USA during 1988–1989 (Costa, Brown, Sivasupramaniam, & Bird, 1993), was highly resistant to pyrethroids, a major factor involved in establishment and displacement of the endemic A mitotype there (Himler et al., 2011), likely occurring following spread from the introduction of *Rickettsia*‐infected *B. tabaci*. Because the Asia II‐1 mitotype occurs in cotton crops experiencing routine insecticide exposure, *Rickettsia* OTUs may at some point be found fixed in Pakistan, a scenario that could involve a forewarning of an unexpected increase in Asia II‐1 insecticide susceptibility (Ghanim & Kontsedalov, 2009; Kontsedalov et al., 2008).

Finally, the frequency of *Hemipteriphilus* (Bing et al., 2013) was greater in Asia II‐7 (*n* = 23) than Asia II‐1 (*n* = 250) mitotypes, at a 39% and 6% frequency, respectively. The relatively high abundance of *Hemipteriphilus* in Lahore, where urban landscapes are abundant and pesticide use is rare, suggests this endosymbiont may have an important role in mitotype adaptability to this and perhaps other micro‐environmental niches.

Together, these results highlight the importance of routine monitoring of endosymbiont OTUs and the associated *B. tabaci* mitotypes that could greatly improve whitefly management strategies by associating OTU assemblages, mitotypes, and CLCuD‐begomoviral species and strains with outbreaks. Routine surveillance and mapping would provide an early warning system such that precautions associated with the different management practices suspected of exacerbating CLCuD outbreaks, including shifting cotton genotypes with no knowledge of resistance to specific virus strain or species, and insecticide programs that stimulate whitefly upsurgences among particular mitotypes or others.

## 5 CONCLUSIONS

The primary endosymbiont *Portiera* and secondary endosymbiont *Arsenophonus* were fixed in the Asia II populations. In contrast, *Cardinium* was detected in 29% of *B. tabaci* sampled, whereas *Hemipteriphilus*, *Rickettsia,* and *Wolbachia* were detected rarely, making them minor endosymbionts, at a 2%–8% frequency. Collectively, at least 43 OTUs taxonomically classified bacterial genera comprised the microbiome of the three Asia II whitefly mitotypes.

The results predict horizontal transfer has occurred for several unique OTUs of *Arsenophonus*, *Cardinium*, *Hemipteriphilus,* and *Wolbachia* between the Asia II mitotypes where their ranges overlapped in Lahore and that certain *Arsenophonus* OTUs were uniquely present in one mitotype over the other. Also, based on the high fixation rate of *Arsenophonus*, it is tempting to speculate the occurrence of a selective sweep by the widely prevalent OTU assemblage in parallel with/leading up to the recent “recovery” observed for Asia II‐1 in the cotton agroecosystems (Ashfaq et al., 2014; Paredes‐Montero et al., 2019). At the same time, the genetic and geographic expansion of *B. tabaci* populations throughout the Punjab province is consistent with the widespread leaf curl disease outbreak caused by the resistance‐breaking CLCuKoV‐Bur strain that affected much of the cotton crop (to ~2016), immediately before and during the time this study was conducted.

Although somewhat speculative, the whitefly expansion may also have contributed to the virus outbreak if the Asia II‐1 is found to be a preferentially competent vector of CLCuKoV‐Bur, over other known vector mitotype–begomoviral combinations of CLCuD species or strains in the Punjab (Pan, Cui, & Chen, 2018). This scenario is further consistent with a role of *Cardinium* and/or *Rickettsia* in the geographic and genetic demographic expansions resulting from an increase in female offspring production concurrent with enhanced insecticide resistance, respectively (Caballero, 2007).

Finally, also of interest is that the composition of OTU assemblages that associate with different mitotypes can provide a useful tool and indicator of genetic/genomic relatedness among the Asia II *B. tabaci* mitotypes studied here and also for future studies of large‐scale relationships among global mitotypes.

## CONFLICT OF INTEREST

The authors declare no conflict of interest.

## AUTHOR CONTRIBUTIONS

J.R.P.‐M. performed analyses, wrote the first draft, and contributed to methodology, review and editing. M.Z.‐U.‐R., U.H., and M.S.H. conducted fieldwork, collected data, contributed to methodology, and data curation. H.‐W.H. contributed to conceptualization, methodology and review. J.K.B. conceived the ideas, and contributed to methodology, resources, funding acquisition, supervision, review and editing.

## Data Availability

The mt*COI* and *16S rDNA* sequences were deposited in GenBank, and accessions numbers are available in Table S2. The raw data used to estimate symbionts frequency and distributions, as well as the samples collection information (Figures [Link], [Link], [Link] and Tables [Link], [Link], [Link], [Link]), were deposited to Dryad at the following URL: https://doi.org/10.5061/dryad.gxd2547gs.
