# Supplementary material for: Genetic variability, community structure, and horizontal transfer of endosymbionts among three Asia II‐Bemisia tabaci mitotypes in Pakistan
Source: Ecol Evol. 2020 Feb 12;10(6):2928–43. doi: 10.1002/ece3.6107 (PMC7083670; doi:10.1002/ece3.6107)

Figure S1. Bayesian phylogeny of the *Bemisia tabaci* mitotypes from Pakistan based on the 3'-end fragment of the mitochondrial cytochrome oxidase I gene (*COI*) (725 bp). Acronyms are lineages designations by De Moya et al. (2019). Numbers at nodes are posterior probabilities.

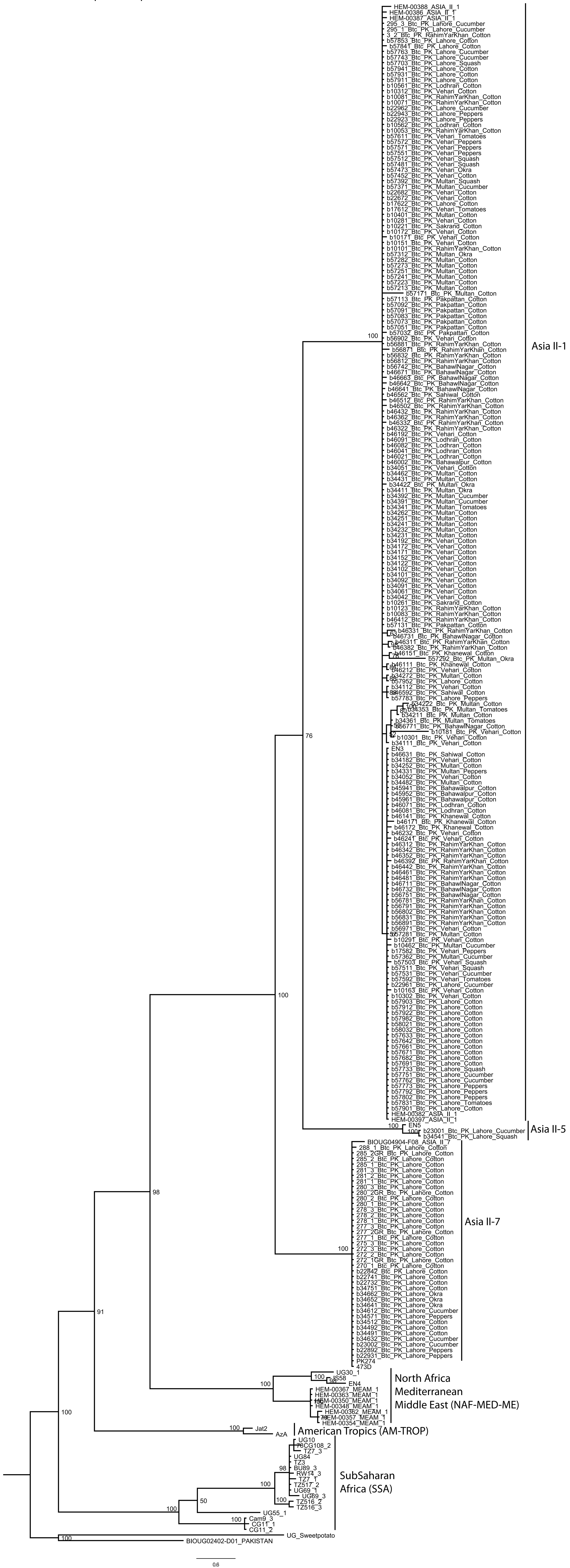

Supplement: Supplementary file 1 [file ECE3-10-2928-s001.pdf]
