# Supplementary material for: Genetic variability, community structure, and horizontal transfer of endosymbionts among three Asia II‐Bemisia tabaci mitotypes in Pakistan
Source: Ecol Evol. 2020 Feb 12;10(6):2928–43. doi: 10.1002/ece3.6107 (PMC7083670; doi:10.1002/ece3.6107)

Figure S2. Distribution of **a** *Portiera,* **b** *Arsenophonus,* **c** *Cardinium,* **d** *Hemipteriphilus,* **e** *Rickettsia,* and **f** *Wolbachia* in twelve districts of Pakistan. Bars represent the relative abundance of endosymbionts per district. *Bemisia tabaci* mitotypes Asia II-5 and -7 were only recorded from Lahore, whereas mitotype Asia II-1 was widely distributed across cotton growing districts. Blue lines in map represent rivers, dotted lines indicate provincial limits and the polygon shows the cotton growing area of Pakistan.

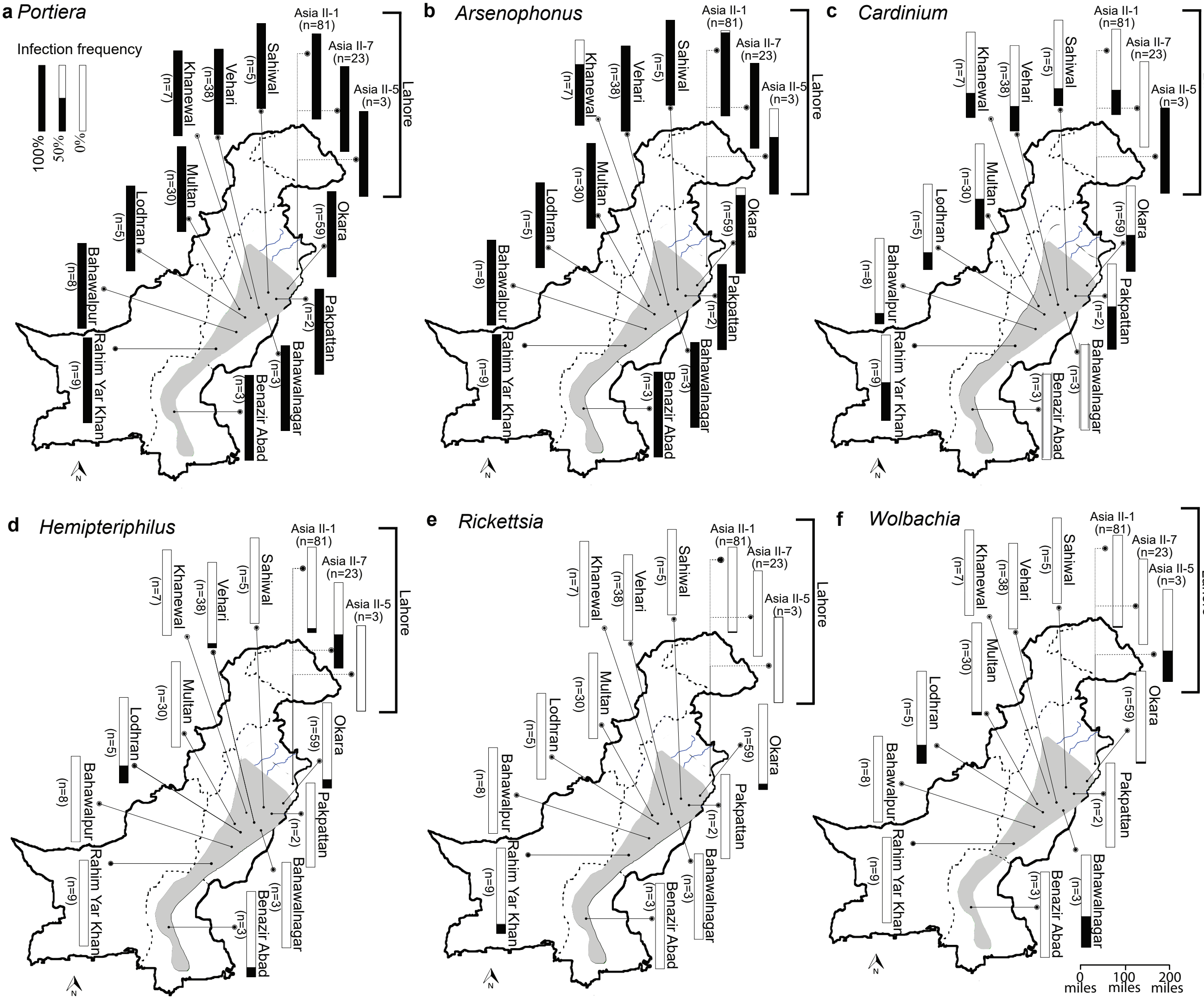

Supplement: Supplementary file 2 [file ECE3-10-2928-s002.pdf]
