# Supplementary material for: Genetic variability, community structure, and horizontal transfer of endosymbionts among three Asia II‐Bemisia tabaci mitotypes in Pakistan
Source: Ecol Evol. 2020 Feb 12;10(6):2928–43. doi: 10.1002/ece3.6107 (PMC7083670; doi:10.1002/ece3.6107)

Figure S3. Rarefaction curves for individual whitefly samples. Red lines are whitefly samples belonging to Asia II-7, black lines are Asia II-1 individuals and blue are whitefly samples from the Asia II-5 group. The curves were generated in mothur v1.43.0 based on the observed richness with 1000 iterations.

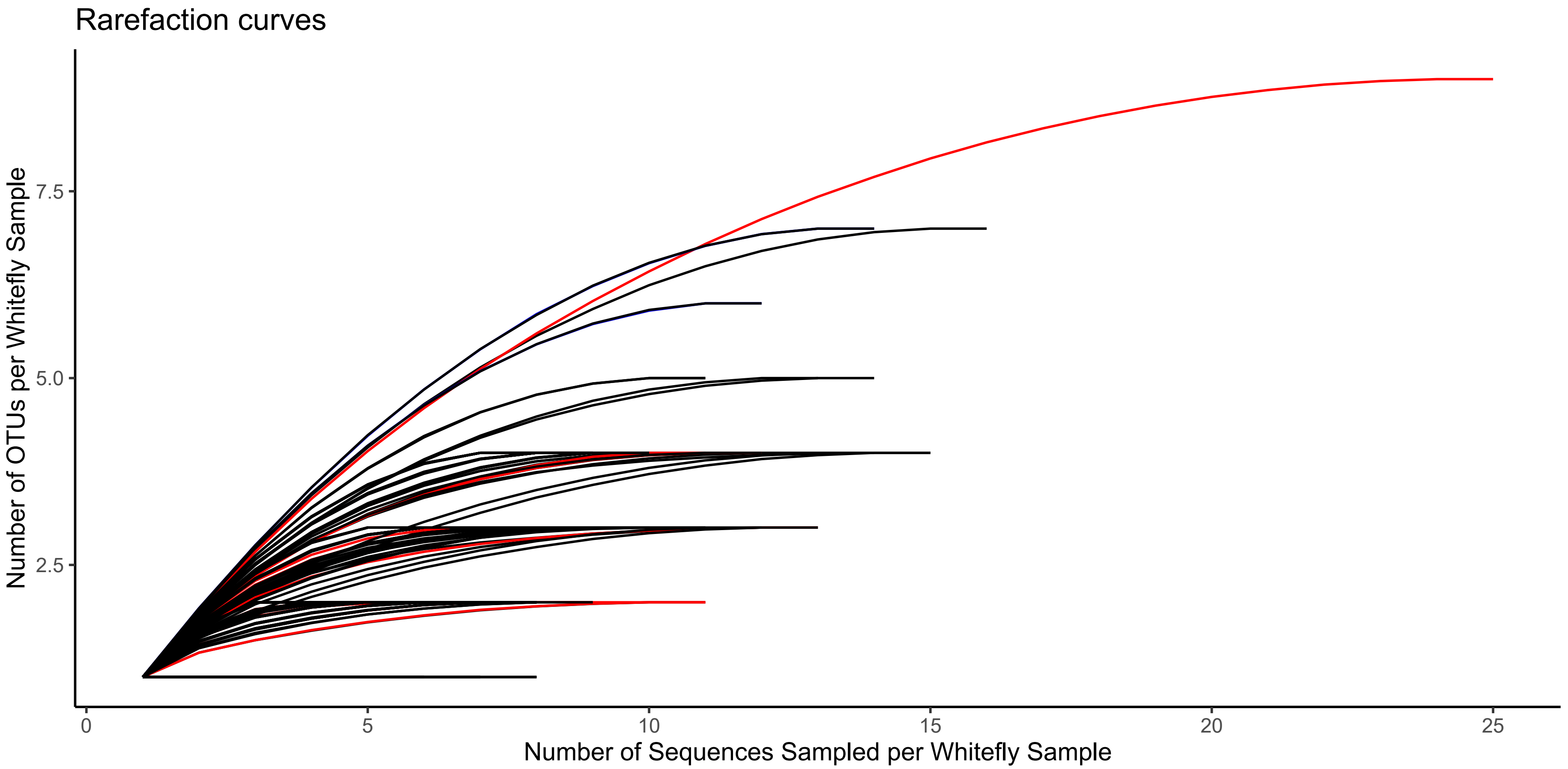

Supplement: Supplementary file 3 [file ECE3-10-2928-s003.pdf]
