# Supplementary material for: Genetic variability, community structure, and horizontal transfer of endosymbionts among three Asia II‐Bemisia tabaci mitotypes in Pakistan
Source: Ecol Evol. 2020 Feb 12;10(6):2928–43. doi: 10.1002/ece3.6107 (PMC7083670; doi:10.1002/ece3.6107)
Supplement: Supplementary file 5 [file ECE3-10-2928-s005.docx]

**Genetic variability, community structure, and horizontal transfer of endosymbionts among three Asia II-*Bemisia tabaci* mitotypes in Pakistan**

**Ecology and Evolution**

Jorge R. Paredes-Montero, Muhammad Zia-Ur-Rehman, Usman Hameed, Hans-Werner Herrmann, Muhammad Saleem Haider, and Judith K. Brown

**Corresponding Author:** Judith .K. Brown

School of Plant Sciences

University of Arizona

Tucson, AZ 85721 USA

jbrown@ag.arizona.edu

(520)-621-1402

**Table S2.** Taxonomic classification, accession numbers of *16S ribosomal RNA* (*16S rRNA*) sequences and host mitochondrial *cytochrome oxidase I* (*mtCOI*), sample location, whitefly host plant, and relative frequency per mitotype of operational taxonomic units (OTUs) detected in whitefly samples from Pakistan

| OTU Number | Taxonomy | 16S rRNA Accession Number | Host mtCOI Accession Number | Location | Isolation Source | Relative Frequency | | |
| --- | --- | --- | --- | --- | --- | --- | --- | --- |
|  |  |  |  |  |  | ASIA II-1 | ASIA II-5 | ASIA II-7 |
| Otu0001 | Portiera | MN187319 | KT708828 | Lahore | Squash | 781 | 8 | 66 |
| Otu0002 | Arsenophonus | MN187320 | KT708826 | Lahore | Pepper | 187 | 0 | 0 |
| Otu0003 | Arsenophonus | MN187321 | KT708836 | Lahore | Cotton | 494 | 0 | 69 |
| Otu0004 | Cardinium | MN187322 | KT708826 | Lahore | Tomato | 160 | 6 | 0 |
| Otu0005 | Hemipteriphilus | MN187323 | KT708857 | Lahore | Cotton | 44 | 0 | 39 |
| Otu0006 | Shigella | MN187324 | KT708829 | Lahore | Okra | 43 | 0 | 4 |
| Otu0007 | Acinetobacter | MN187325 | KT708826 | Multan | Cotton | 35 | 0 | 0 |
| Otu0008 | Rickettsia | MN187326 | KT708828 | Lahore | Cotton | 17 | 0 | 0 |
| Otu0009 | Pseudomonas | MN187327 | KT708826 | Pakpattan | Cotton | 17 | 0 | 0 |
| Otu0010 | Wolbachia | MN187328 | KT708834 | Lahore | Pepper | 12 | 2 | 0 |
| Otu0011 | Ralstonia | MN187329 | KT708941 | Lahore | Cotton | 6 | 0 | 0 |
| Otu0012 | Lactobacillus | MN187330 | KT708836 | Lahore | Cotton | 8 | 0 | 0 |
| Otu0013 | Rhizobium | MN187331 | KT708890 | Multan | Okra | 4 | 0 | 0 |
| Otu0014 | Propionibacterium | MN187332 | KT708900 | Lahore | Cucumber | 32 | 0 | 10 |
| Otu0015 | Staphylococcus | MN187333 | KT708836 | Lahore | Cotton | 4 | 0 | 0 |
| Otu0016 | Sediminibacterium | MN187334 | KT708826 | Khanewal | Cotton | 4 | 0 | 0 |
| Otu0017 | Achromobacter | MN187335 | KT708826 | Pakpattan | Cotton | 4 | 0 | 0 |
| Otu0018 | Novosphingobium | MN187336 | KT708826 | Lahore | Cucumber | 2 | 0 | 0 |
| Otu0019 | Arsenophonus | MN187337 | KT708840 | Lahore | Cucumber | 0 | 4 | 4 |
| Otu0020 | Aquabacterium | MN187338 | KT708826 | Khanewal | Cotton | 4 | 0 | 0 |
| Otu0021 | Streptococcus | MN187339 | KT708828 | Lahore | Pepper | 4 | 0 | 0 |
| Otu0022 | Bradyrhizobium | MN187340 | KT709079 | Lahore | Pepper | 2 | 0 | 0 |
| Otu0023 | Janthinobacterium | MN187341 | KT708840 | Lahore | Cucumber | 0 | 0 | 2 |
| Otu0024 | Arsenophonus | MN187342 | KT708854 | Lahore | Squash | 0 | 4 | 0 |
| Otu0025 | Ochrobactrum | MN187343 | KT708941 | Vehari | Cucumber | 4 | 0 | 0 |
| Otu0026 | Microbacterium | MN187344 | KT708826 | Lahore | Pepper | 4 | 0 | 0 |
| Otu0027 | Comamonas | MN187345 | KT708826 | Lahore | Cotton | 2 | 0 | 0 |
| Otu0028 | Methylophilus | MN187346 | KT708826 | Multan | Cotton | 2 | 0 | 0 |
| Otu0029 | Brachybacterium | MN187347 | KT708828 | Lahore | Squash | 2 | 0 | 0 |
| Otu0030 | Rhizobiaceae | MN187348 | KT708829 | Multan | Okra | 2 | 0 | 0 |
| Otu0031 | Methylobacterium | MN187349 | KT708839 | Lahore | Cotton | 0 | 0 | 2 |
| Otu0032 | Iamia | MN187350 | KT708924 | Lahore | Cotton | 2 | 0 | 0 |
| Otu0033 | Rubrobacter | MN187351 | KT708826 | Lahore | Cotton | 2 | 0 | 0 |
| OTU Number | Taxonomy | 16S rRNA Accession Number | Host mtCOI Accession Number | Location | Isolation Source | Relative Frequency | | |
|  |  |  |  |  |  | ASIA II-1 | ASIA II-5 | ASIA II-7 |
| Otu0034 | Nesterenkonia | MN187352 | KT708839 | Lahore | Tomato | 0 | 0 | 2 |
| Otu0035 | Chitinophagaceae | MN187353 | KT708832 | Vehari | Cotton | 2 | 0 | 0 |
| Otu0036 | Bacillus | MN187354 | KT708826 | Lahore | Cotton | 2 | 0 | 0 |
| Otu0037 | Desemzia | MN187355 | KT708923 | Lahore | Cotton | 2 | 0 | 0 |
| Otu0038 | Olivacter | MN187356 | KT708836 | Lahore | Cotton | 2 | 0 | 0 |
| Otu0039 | Lactococcus | MN187357 | KT708840 | Lahore | Cucumber | 0 | 0 | 2 |
| Otu0040 | Arsenophonus | MN187358 | KT708839 | Lahore | Cotton | 0 | 4 | 0 |
| Otu0041 | Arsenophonus | MN187359 | KT708840 | Lahore | Cucumber | 0 | 4 | 5 |
| Otu0042 | Brevundimonas | MN187360 | KT708828 | Lahore | Squash | 4 | 0 | 0 |
| Otu0043 | Stenotrophomonas | MN187361 | KT708826 | Pakpattan | Cotton | 4 | 0 | 0 |
